# Experimental and Theoretical Investigation of Thiazolyl Blue as a Corrosion Inhibitor for Copper in Neutral Sodium Chloride Solution

**DOI:** 10.3390/ma11061042

**Published:** 2018-06-19

**Authors:** Li Feng, Shengtao Zhang, Yujie Qiang, Yue Xu, Lei Guo, Loutfy H. Madkour, Shijin Chen

**Affiliations:** 1 School of Chemistry and Chemical Engineering, Chongqing University, Chongqing 400044, China; fengli_cqu@163.com (L.F.); yqiang_cqu@163.com (Y.Q.); cqtanjc@163.com (Y.X.); 2 Material Corrosion and Protection Key Laboratory of Sichuan province, Zigong 643000, China; 3 School of Material and Chemical Engineering, Tongren University, Tongren 554300, China; 4 Chemistry Department, Faculty of Science and Arts, Al Baha University, Baljarashi 65635, Saudi Arabia; loutfy_madkour@yahoo.com; 5 Bomin Electronics Ltd., Meizhou 514021, China; fwjshen@163.com

**Keywords:** corrosion, copper, electrochemistry, XPS, molecular simulation

## Abstract

The anticorrosion effect of thiazolyl blue (MTT) for copper in 3% NaCl at 298 K was researched by electrochemical methods, scanning electron-microscopy (SEM), and atomic force microscopy (AFM). The results reveal that MTT can protect copper efficiently, with a maximum efficiency of 95.7%. The corrosion inhibition mechanism was investigated by X-ray photoelectron spectroscopy (XPS), Fourier transform infrared spectral (FT-IR), and theoretical calculation. The results suggest that the MTT molecules are adsorbed on metal surface forming a hydrophobic protective film to prevent copper corrosion. It also indicates that the MTT and copper form covalent bonds. The molecular dynamic simulation further gives the evidence for adsorption. The adsorption isotherm studies demonstrate that a spontaneous, mixed physical and chemical adsorption occurs, which obeys Langmuir adsorption isotherm. The present research can help us better understand the corrosion inhibition process and improve it.

## 1. Introduction

Because of excellent performance, copper and copper alloys are widely used in many industries including electrical, machinery, transportation and marine [1,2,3]. However, the exposure of copper has increased opportunities for corrosion and destruction. Especially in marine environments, the damage is particularly severe because of chloride ions [4,5,6,7,8,9,10,11]. It is worth noting that copper corrosion causes huge economic losses, energy losses, and safety issues. Therefore, it is significant and necessary to study corrosion inhibition in a neutral chloride solution. Furthermore, understanding the inhibition mechanism will help us to improve inhibition efficiency.

Currently, various methods have been used to inhibit the corrosion of copper and its alloys [12,13,14,15,16]. One of the most traditional and efficient methods uses organic matter as a corrosion inhibitor [17,18,19]. In recent years, many organics, including benzotriazole (BTA), thiazole, iminazole, pyrrodiazole and their derivatives, are used as inhibitors [20,21,22,23,24]. However, most organic compounds containing nitrogen, oxygen, phosphorus, and sulfur are difficult to dissolve in aqueous solution, and can also cause huge damage to environment. Considering toxicity and water solubility, the development of novel, environment-friendly corrosion inhibitors is urgent. Ionic liquids as green compounds have attracted much attention because of their excellent properties. They are characterized by low toxicity, large electrochemical window, negligible vapor pressure, non-volatility, non-flammability, easy degradability, easily recyclability, and easily solubility in water. It has been reported that ionic liquids inhibit the corrosion of steel, but there is little research on copper [25,26,27,28,29]. In addition, the inhibition mechanism of ionic liquids is unclear. Based on the above considerations, this study was conducted.

Thiazolyl blue (MTT) composed with an organic cation and a bromide anion is expected to have a good corrosion resistance performance. The aim of this study is to research the corrosion inhibition performance and discuss the inhibition mechanism of thiazolyl blue (MTT) for copper in simulated seawater solution (3% NaCl) at 298 K. The inhibition efficiency was studied by electrochemical measurements. The surface roughness and morphology of copper are observed by atomic force microscopy (AFM) and scanning electron-microscopy (SEM). The inhibition mechanism was further researched by Fourier transform infrared spectral (FT-IR), X-ray photoelectron spectroscopy (XPS), and molecular dynamics simulation (MD). It is found that the MTT molecules form covalent bonds with copper by N and S atoms. The adsorption model is parallel from MD, which is the largest area of coverage. The adsorption forms a protective film that insulates the metal surface from the corrosion medium. Thus, the MTT shows excellent corrosion resistance. The present work gives us an in-depth understanding of the inhibition principles of corrosion inhibitor, which is significant to inhibit copper corrosion.

## 2. Materials and Experimental

### 2.1. Materials

**Figure 1:**
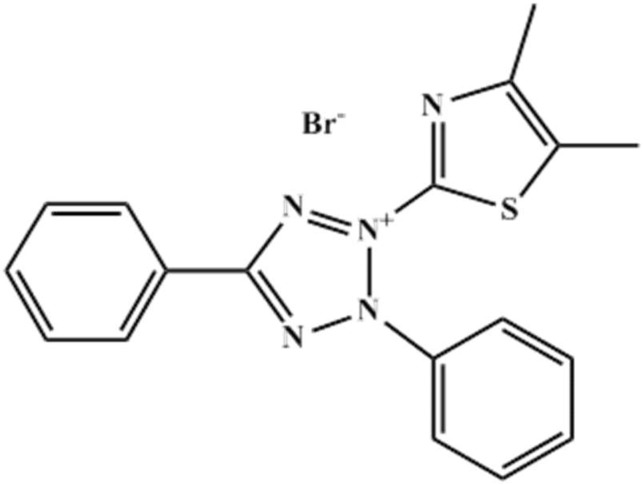
The Molecular structure of thiazolyl blue (MTT).

Thiazolyl blue (MTT) (C_18_H_16_BrN_5_S, Aladdin, 98.0%, Shanghai, China, as shown in Figure 1, and sodium chloride (Aladdin, 99.5%, Shanghai, China) were used without any purification. The test solution was 3% NaCl in the presence and absence of various MTT (0.05 mM, 0.2 mM, 0.5 mM, 1.0 mM and 5.0 mM). In contrast, the blank solution was only 3% NaCl. Before each experiment, the sample was polished with different sandpapers (400, 800, 1200, 2000, Lifeng Inc., Huizhou, China), then cleaned ultrasonically with distilled water and alcohol respectively, before drying. The temperature was controlled by an aqueous thermostat (Lichen Inc., Shanghai, China). The studied metal was pure copper (more than 99.5% Cu).

### 2.2. Electrochemical Measurements

The electrochemical workstation (CHI660C, Chenhua, Shanghai, China) was applied for the electrochemical experiments by a typical three-electrode system at 298 K. This electrode system consisted of a working electrode (WE, pure copper), the reference electrode (SCE, saturated calomel electrode), and counter electrodes (CE, platinum electrode). The area of the working electrode was 1 cm^2^. At first, the copper electrode was at open circuit potential (OCP) for 1800 s to achieve a stable state. For electrochemical impedance spectroscopy (EIS) tests, the excitation signal amplitude was 5 mV, the potential was OCP, and the range of frequency was 10^−2^~10^5^ Hz. Finally, the potentiodynamic polarization curves were performed with a scan rate of 0.5 mV/s, and the potential range of ±250 mV versus the OCP. The inhibition efficiencies (*η*) from EIS and the polarization curves were calculated by (1) and (2) [30].

(1) $$\eta \;=\;\frac{R_{p}\;-\;R_{p,0}}{R_{p}}\;\times \;100\%$$

(2) $$\eta \;=\;\left(\frac{I_{\mathrm{corr},0}\;-\;I_{\mathrm{corr}}}{I_{\mathrm{corr},0}}\right)\;\times \;100\%$$

Here, *I*_corr_ and *I*_corr,0_ represent corrosion current density, and *R*_p_ and *R*_p,0_ represent polarization resistance with and without inhibitors. Besides, a durability test was performed, and the concrete results can be found in Figure S (see the Supplementary Materials).

### 2.3. Surface Analysis

The AFM (MFP-3D-BIO, Asylum Research, Goleta, CA, USA), the contact angle (JC2000C1, Zhongchen, Shanghai, China), and SEM (JSM-7800F, JEOL Ltd., Tokyo, Japan) were applied to further investigate copper appearance. The X-ray photoelectron spectroscopy (XPS, PHI 5700, Ulvac-Phi, Chigasaki, Japan) and the FT-IR (BRUKER, Bremen, Germany) measurements were employed to research the inhibition mechanism. The dimensions of the sample were 0.5 cm × 0.5 cm × 0.5 cm for SEM, and 1.00 cm × 1.00 cm × 0.1 cm for AFM, XPS, and FT-IR. Prior to each test, the specimens were immersed in a blank solution (3% NaCl) and 5 mM MTT test solution respectively for 24 h at 298 K before measurements. The contact angles were tested using the sessile water drop method. The XPS measurement was conducted with Al Kα X-ray source, and analyses were at an emitted photoelectron take-off angle of 90°. The binding energy scale was adjusted by the C1s of 284.8 eV and the XPS Peak 4.1 software was used to de-convolve all peaks using the Shirley-type background. For FT-IR, the pure substance (MTT) was compared with Cu-MTT film.

### 2.4. Calculation Methods

The theoretical calculations were carried out by Materials Studio software 8.0 (BIOVIA Inc., San Diego, CA, USA) to further research the inhibition mechanism. The quantum chemical calculations were achieved with DMol^3^ module. The molecular structure was geometrically optimized with the density functional theory (DFT) and the B3LYP functional. The related parameters were obtained including dipole moment (*μ*), the lowest unoccupied molecular orbital (*E*_LUMO_), the energy of highest occupied molecular orbital (*E*_HOMO_), and the energy of and energy gap (Δ*E*_Cu-inhibitor_ = *E*_LUMO_ − *E*_HOMO_). In addition, Forcite module was used to conduct the MD. The adsorption process between MTT cation and the Cu (111) surface was simulated in the aqueous phase with NVT ensemble, 1000 ps simulation time, and 1.0 fs time step at 298 K. The adsorption energy (*E*_Cu-inhibitor_) was calculated and analyzed by the equation [31].

(3) $$E_{\mathrm{Cu}-\mathrm{inhibitor}}\;=\;E_{\mathrm{Total}}\;-\;(E_{\mathrm{inhibitor}}\;+\;E_{\mathrm{Cu}})$$

## 3. Results and Discussion

### 3.1. EIS Analysis

**Figure 2:**
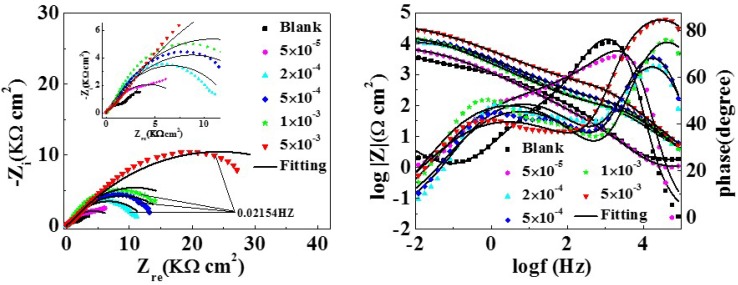
Nyquist and bode plots for the copper electrode with and without different concentrations of MTT in 3% NaCl at 298 K.

**Figure 3:**
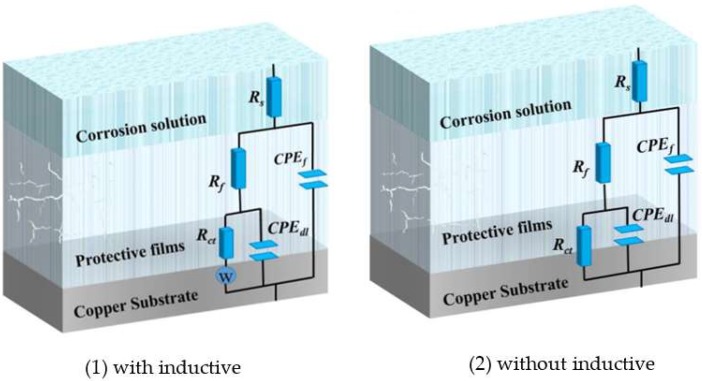
The equivalent circuits with and without inductive.

The EIS test was carried out to get information on corrosion inhibition performance. Figure 2 displays the Nyquist and bode curves with and without various concentrations of MTT for copper electrodes. The equivalent circuit models fitting the EIS experimental data are shown in Figure 3, and relevant parameters are shown in Table 1.

In Figure 3, the original lines are consistent with the fitting lines. Obviously, the Nyquist plots of the black and the lower concentration include a straight line at low frequencies and an oblate capacitive loop at high frequencies. According to corrosion resistance process, the capacitive loop attributes to the charge transfer process (*R*_ct_) and the double layer behavior (*C*_dl_), where the dispersing effect results in the oblate semi-circle. In addition, the straight line at low frequency is the warburg impedance (*W*), which is related to the mass transportation between the copper surface and the bulk solution or the diffusion of oxygen [32]. However, the inductive loop disappears as the increase of MTT, indicating that the corrosion of copper is controlled by the charge transfer process [33]. It can be observed that the diameter of the semicircle increases with addition of MTT concentration, which suggests the inhibition performance is improved by the increase of MTT in an aggressive medium at 298 K. For the analysis of bode plots, the impedance value also increases for the whole frequency range. This phenomenon demonstrates that the inhibition performance increases with the augment of MTT. In addition, the shape of phase plots has changed: the two time constants are presented with the addition of MTT, leading us to speculate that the process of corrosion had changed [34]. It can be accepted that the MTT molecules form a protective film on copper by adsorption. Comparing the blank and MTT-modified electrodes, the phase angle at the high frequency is greater than 0 and closer to 90, which also indicates a protective film has formed on the copper surface [35].

For equivalent circuits, *R*_p_ is the polarization resistance (*R*_p_ = *R*_ct_ + *R*_f_), *R*_f_ represents the film resistance formed on the copper electrode surface, *R*_ct_ is the charge transfer resistance, *Q*_1_ and *Q*_2_ present the constant phase elements (CPE), *C*_f_ and *C*_dl_ are film capacitance and double-layer capacitance respectively. The CPE often represents pure capacitor, which is calculated from the following Equation (4) [36,37]:

(4) $$Z_{\mathrm{CPE}}\;=\;\frac{1}{Y{\left(j\omega \right)}^{n}}$$

Here, *w* is the angular frequency, n is the deviation parameter, *Y* is the CPE constant, and *j* is the imaginary root.

In Table 1, the values of *C*_dl_ and *C*_f_ decrease with the increase of MTT, which can be explained by Equations (5) and (6) [38]:

(5) $$C_{\mathrm{dl}}\;=\;\frac{\varepsilon^{0}\varepsilon }{d}S$$

(6) $$C_{f}\;=\;\frac{F^{2}S}{4\mathrm{RT}}$$

where *S* is the surface area of the electrode exposed to the corrosive solution, *F* is the Faraday’s constant, *ε*^0^ is the permittivity constant of the air, *ε* is the local dielectric constant of the film, and *d* is the thickness of the electric double layer. As more corrosion inhibitor molecules replace water molecules occupying the active sites with the increase of MTT concentration, which cause distinct electric double layer thickness increases, the area of copper electrode exposed to corrosion solution and local dielectric constant (*ε*) decreases [39]. The values of *R*_ct_ and *R*_p_ both have a reverse change, suggesting that the corrosion reaction is inhibited effectively. At the same time, the corrosion inhibition efficiency (*η*) increased with inhibitor concentration, the maximum efficiency is 95.73%. Furthermore, the durability of the Cu-MTT film is stable over a short period of time (<5 h) from durability tests. However, when the immersion time is more than 10 h, the protective effect of the film decreases slightly. This is due to corrosive substances in the solution, such as Cl^−^, or unsound film.

### 3.2. Potentiodynamic Polarization Curves Analysis

**Figure 4:**
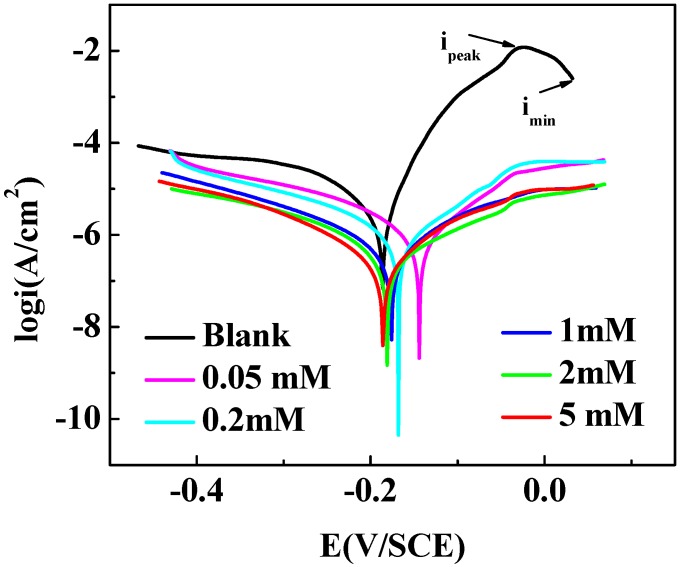
Polarization curves for copper with and without different concentrations of MTT in 3% NaCl at 298 K.

Figure 4 is the potentiodynamic polarization curves (Tafel) with and without different concentrations of MTT for copper in 3% NaCl at 298 K. The relevant parameters are obtained in Table 2. The cathodic reaction on copper surface in NaCl solution is Equation (7) [40]:

(7) $$O_{2}\;+\;4e^{-}\;+\;2H_{2}O\;\to \;4{\mathrm{OH}}^{-}$$

The anodic corrosion reaction follows (8)–(12) [41]:

(8) $$\mathrm{Cu}\;\to \;{\mathrm{Cu}}^{+}\;+\;e^{-}$$

(9) $${\mathrm{Cu}}^{+}\;+\;{\mathrm{Cl}}^{-}\;\to \;\mathrm{CuCl}$$

(10) $$\mathrm{CuCl}\;+\;{\mathrm{Cl}}^{-}\;\to \;{{\mathrm{CuCl}}_{2}}^{-}$$

(11) $${{\mathrm{CuCl}}_{2}}^{-}\;\to \;{\mathrm{Cu}}^{2+}\;+\;2{\mathrm{Cl}}^{-}\;+\;e^{-}$$

(12) $${\mathrm{Cu}}^{2+}\;+\;\mathrm{Cu}\;+\;2{\mathrm{Cl}}^{-}\;\to \;2\mathrm{CuCl}$$

In Figure 4, the maximum current density value (*i*_peak_) is presented because of the corrosion of copper into Cu^+^, as shown in Equation (8). As Equation (9) mentioned, the CuCl film is formed on copper surface, the current density value shows the *I*_min_, and the corrosion is controlled provisionally. If the film is loose, the corrosion reaction will proceed further in chloride ion solution following Equations (10)–(12). With the addition of an inhibitor, a protective, dense film is formed on the surface of copper and corrosion is inhibited. Comparing with the blank, the values of *I*_corr_ decrease in the presence of MTT, which indicates that the copper corrosion is effectively inhibited. This phenomenon demonstrates that the addition of MTT not only inhibits the dissolution of oxygen, but also controls the corrosion of copper in an aggressive medium. In Table 2, *β*_c_ and *β*_a_ represent cathodic and anodic and Tafel slopes, respectively. The *E*_corr_ values move to a positive direction with a minimum moving value of 5 mM MTT, and all variation values lower than 85 mV; thus, the MTT can be defined as a mixed type inhibitor [42]. The corrosion inhibition ability is improved with the addition of the MTT concentration, which can be explained by the fact that more MTT molecules are absorbed on copper surface occupying the active sites to protect copper.

### 3.3. Morphology Analysis

**Figure 5:**
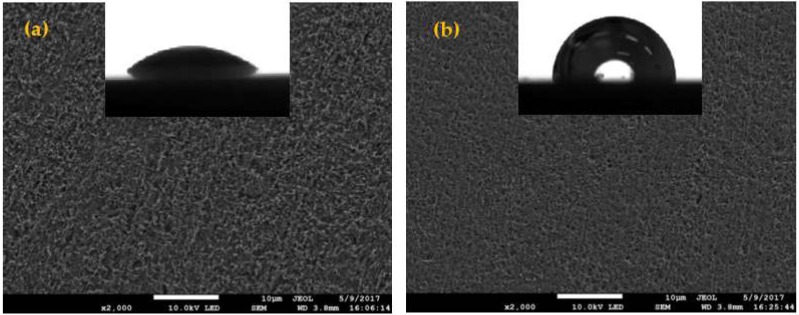
SEM and contact angle morphologies of the copper specimen immersed in 3% NaCl with and without MTT at 298 K ((**a**) the blank, (**b**) 5 mM MTT).

The SEM is considered as an important method for studying surface topography. The SEM and contact angle images of the copper samples with and without MTT are presented in Figure 5. The copper was seriously corroded in blank solution; as shown in Figure 5a, the surface becomes very rough and has many big pits. In contract, the copper surface is less damaged in 5 mM MTT solution (Figure 5b), which indicates that the MTT can prevent the copper from corroding in a neutral chloride solution. The surface of the sample treated with MTT becomes smoother than the blank, also suggesting that the copper is protected efficiently in an aggressive solution. In addition, the contact angle increases from 42.7° for Cu-Blank to 92.8° for Cu-MTT, which also demonstrates that the Cu-MTT film has hydrophobic property. The hydrophobicity gives rise to good corrosion inhibition performance. 

**Figure 6:**
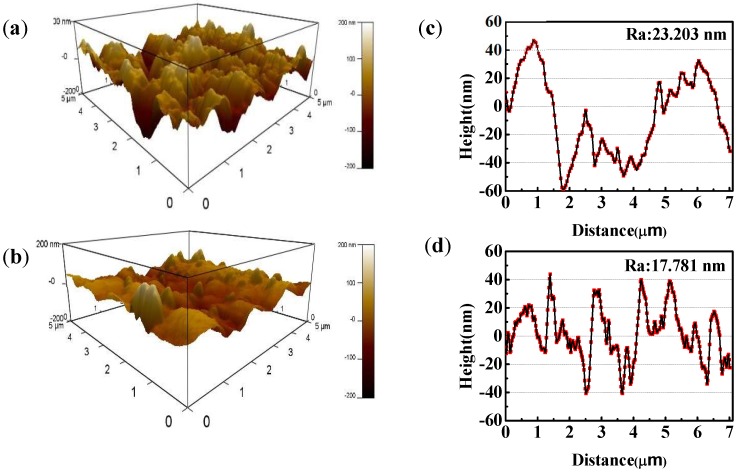
AFM morphologies of the copper specimen immersed in the blank solution with and without 5 mM MTT ((**a**,**c**) the blank, (**b**,**d**) 5 mM MTT).

The AFM 3D images and height plots of copper with and without MTT in 3% NaCl solution at 298 K are showed in Figure 6, which presents more information of copper appearance. Obviously, the blank (Figure 6a) shows a rough structure of the unprotected copper surface with large and deep pits, suggesting that the copper specimens are seriously corroded in a neutral chloride solution. As mentioned in previous reports, chloride ions seriously corrode copper. With the addition of MTT, the copper surface becomes smoother, as shown in Figure 6b, suggesting that MTT can inhibit the attack of corrosive ion to copper. The same conclusion can be drawn from the height plots. The average roughness (*R*_a_) is 23.203 nm for the blank, as shown (Figure 6c), and the values of *R*_a_ reduce to 17.781 nm in the presence of MTT (Figure 6d), which indicates that the MTT can inhibit the copper corrosion in an aggressive medium. These results are in agreement with the above experiments.

### 3.4. FT-IR Spectra

**Figure 7:**
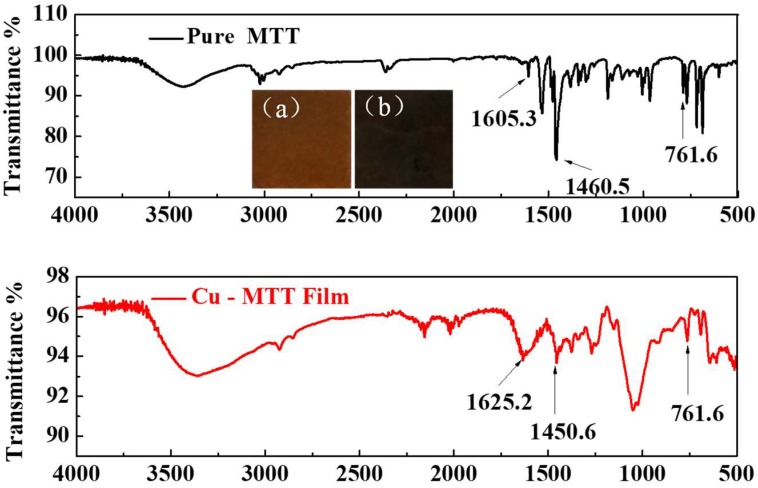
FT-IR spectra of MTT powder and Cu-MTT film. (Insets: morphologies for (**a**) pure copper and (**b**) after immersed in 5 mM MTT).

To get more adsorption information, the FT-IR spectra of Cu-MTT film and pure MTT are compared in Figure 7. From the insets displayed in upper panels of Figure 7, it is obvious that the copper surface shows a darker colored film after immersion. For pure MTT, the bands around 1605.3 cm^−1^, 1460.5 cm^−1^ and 761.6 cm^−1^are attributed to the C=N, C–N and phenyl ring, respectively [43]. There are analogous bands for Cu-MTT film in the same area, suggesting MTT molecules are adsorbed on copper. Furthermore, the bands of C=N and C–N both shift, and the relative adsorption intensity of C–N weakens compared to that of the pure MTT. Maybe there is a chemical interaction between MTT and copper. Then, the clathrate complex is formed on the surface of copper; this passive film can inhibit copper corrosion in aggressive medium. In order to explore the correlation between MTT and copper, the XPS measurement was performed.

### 3.5. XPS Measurements

**Figure 8:**
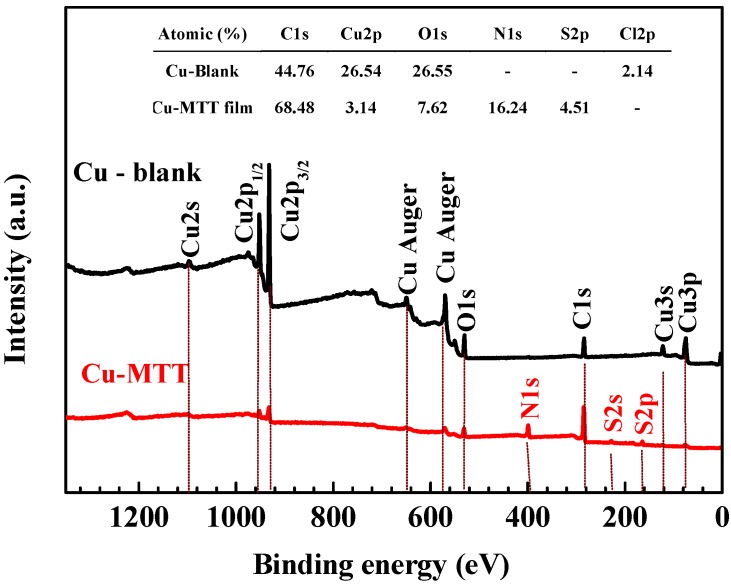
Representative XPS survey spectra for the blank and Cu-MTT film.

**Figure 9:**
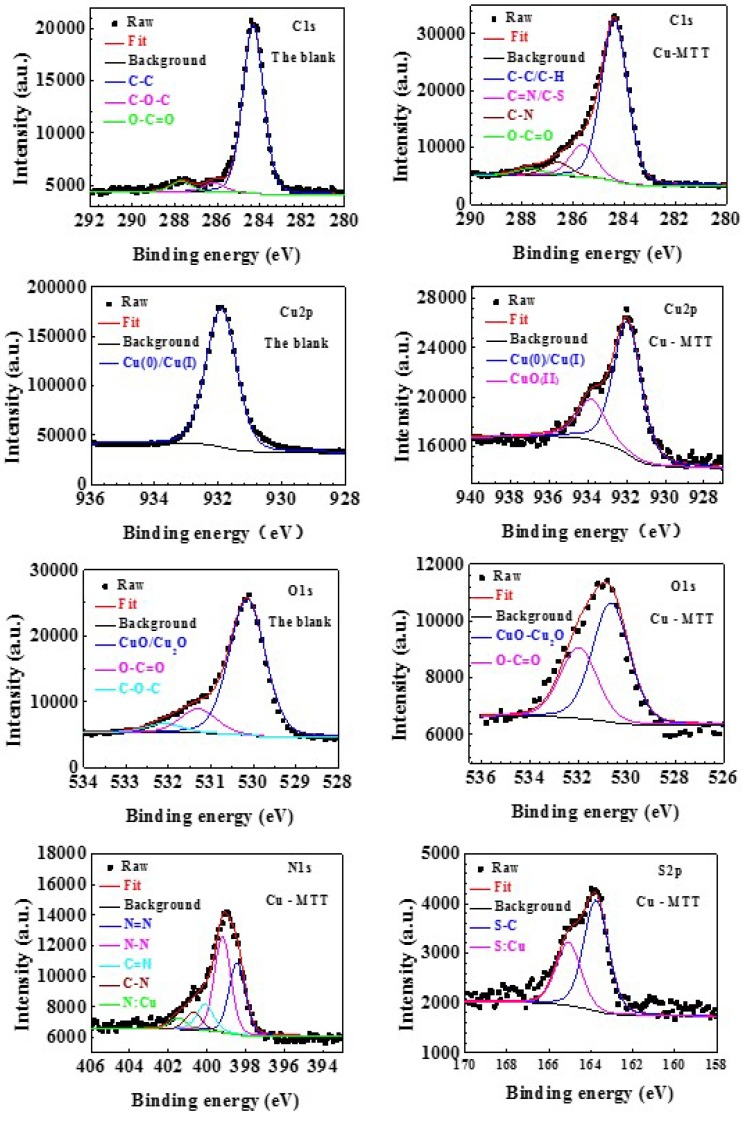
The de-convolution XPS spectra of for the blank and Cu-MTT film, respectively.

The XPS experiment was carried out to study the interaction between copper and MTT. Figure 8 compares the survey spectra for the Cu-blank and Cu-MTT film. C, O, and Cu exist in two samples, and N and S were detected on the Cu-MTT film, but not on the blank copper, which implies that MTT molecules can be absorbed by the copper surface, as mentioned above. Figure 9 shows the de-convolution XPS spectra of the blank (C1s, Cu2p, O1s) and Cu-MTT film (C1s, Cu2p, O1s, N1s and S2p). Correlative binding energy, full width at half maximum (FWHM), and chemical states are listed in Table 3.

In the survey spectra, a small amount of Cl was detected on the Cu-blank due to the CuCl from corrosion, but Cu-MTT was not detected. C, N, and S atomic content increases, suggesting the MTT molecules are absorbed on copper surface. However, O and Cu levels decrease for Cu-MTT, owing to the Cu-MTT film, which indicates the corrosion of copper is effectively inhibited.

For C1s de-convolution of the blank, the peaks at about 284.31 eV, 286.16 eV, and 287.70 eV are attributed to C–C/C–H, C–O–C and O–C=O respectively, which are caused by adventitious carbon pollution [44]. The corresponding peaks can be found in the Cu-MTT film. However, there is an obvious difference between the blank and Cu-MTT. The C=N/C–S (285.62 eV) and C–N (286.61 eV) can be found for Cu-MTT, but not for the blank. This phenomenon demonstrates that MTT molecules are absorbed on the copper surface in a corrosive solution.

It can be observed from the de-convolution spectra of Cu2p_3/2_ that the peaks of Cu(0)/Cu(I) (913.9 eV, 913.95 eV) are contained for two samples. This results suggests that Cu(I) species (CuCl) are the main corrosion products in a neutral chloride solution [45]. Particularly, Cu(II) species are present on the Cu-MTT film, but not for the blank, which is attributed to the corrosion of Cu and the chemical interaction between MTT and copper. Furthermore, the relative intensity of Cu2p_3/2_ for Cu-MTT is smaller compared with the blank, owing to the adsorption of MTT molecules.

For O1s, de-convolution of the blank and Cu-MTT, the O–C=O and C–C–C are due to adventitious oxygen contamination [46]. In Table 3, the lowest peaks, located at 530.14 eV, 530.65 eV, are copper oxide/cuprous oxide (CuO/Cu_2_O) for the two copper samples [47]. The intensity of oxygen reduces because of the addition of MTT, suggesting the oxide of copper is restrained.

The spectra of N1s for Cu-MTT are decomposed into the five peaks in Figure 9, suggesting five chemical forms of N atoms are presented on the copper surface. The type of peak at about 401.77 eV is the result of N:Cu from previous reports [48]. As shown in Table 3, four other peaks represent the type of MTT molecules [49]. As is well known, the nitrogen atom has a pair of lone pair electrons, which could be accepted by the unoccupied molecular orbital of Cu. Thus, it is commonly acceptable that the MTT are absorbed onto the copper surface by N atoms, which is in agreement with our FT-IR spectra analysis. For the spectra of S2p, the Cu-MTT sample contains two chemical states of S; the higher binding energy (165.07 eV) derived from S:Cu, which is a chemical bond between MTT and Cu [50]. The peak of S2p located at 163.75 eV corresponds to S–C from MTT, which also manifests that the MTT is adsorbed on copper surface. It can be concluded that MTT molecules are absorbed chemically onto the copper surface by N and S atoms.

### 3.6. Adsorption Isotherm Analysis

**Figure 10:**
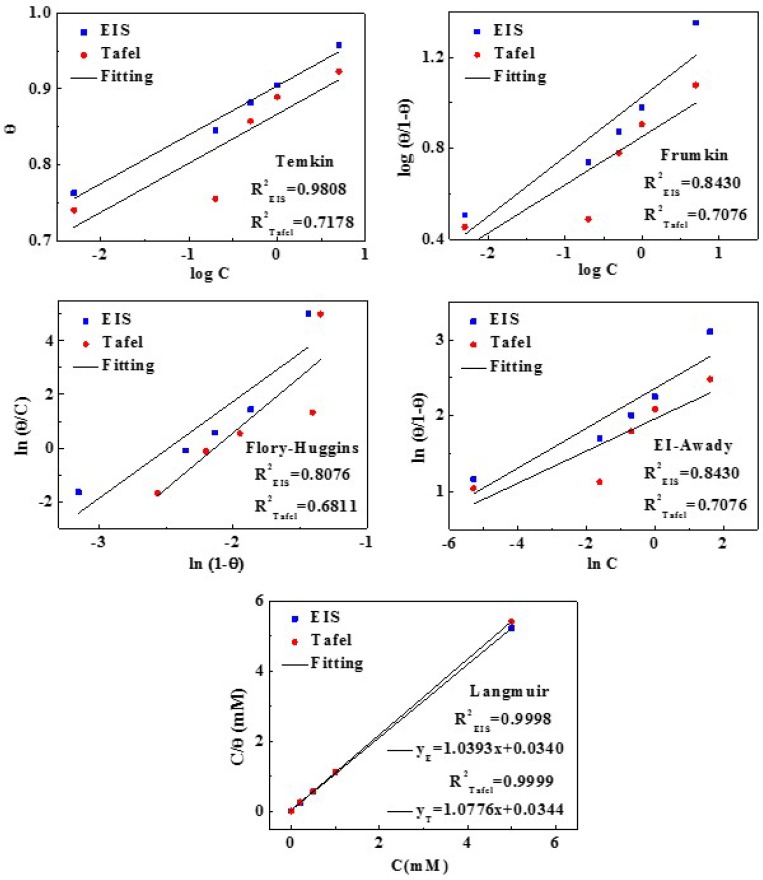
The various adsorption models fitting of MTT on copper surface.

In order to further study the adsorption process of inhibitor molecules on copper surface, various adsorption isotherms were applied to fit experimental data from Tafel and EIS data, as shown in Figure 10. The form of these isotherms is listed (13)−(17):

(13) Temkin adsorption isotherm: KC = exp(2aθ)

(14) $$\mathrm{Frumkin}\;\mathrm{adsorption}\;\mathrm{isotherm}:\;\ln\left[\frac{\theta }{\left(1-\theta \right)C}\right]\;=\;\mathrm{lnK}\;+\;2a\theta$$

(15) $$\mathrm{Flory}-\mathrm{Huggins}\;\mathrm{adsorption}\;\mathrm{isotherm}:\;\ln\frac{\theta }{C}\;=\;\mathrm{xln}\left(1-\theta \right)\;+\;\ln\left(xK_{\mathrm{ads}}\right)$$

(16) $$\mathrm{EI}-\mathrm{Awady}\;\mathrm{adsorption}\;\mathrm{isotherm}:\;\ln\frac{\theta }{1-\theta }\;=\;\mathrm{ylnC}\;+\;{\mathrm{lnK}}^{,}$$

(17) $$\mathrm{Langmuir}\;\mathrm{adsorption}\;\mathrm{isotherm}:\;\frac{C}{\theta }\;=\;\frac{1}{K_{\mathrm{ads}}}\;+\;c$$

For the adsorption behavior of MTT, the best description is Equation (17) named Langmuir adsorption. Here, *θ* is the surface coverage equaling the corrosion inhibition efficiency, and *C* is the inhibitor concentration. All linear regression coefficients (*R*^2^) are clothing to 1, and all fitted results are in good agreement with the Langmuir adsorption. The relevant thermodynamic parameters are obtained in Table 4. The Δ*G*^0^_ads_ is calculated by the Equation (18) [51]:

(18) $$K_{\mathrm{ads}}\;=\;\frac{1}{55.5}\exp\left(\frac{-∆G_{\mathrm{ads}}^{0}}{\mathrm{RT}}\right)$$

Generally speaking, a low value of Δ*G*^0^_ads_ and a high value of *K*_ads_ manifest that inhibitors can be absorbed on metal strongly, showing better inhibitive behavior. The Δ*G*^0^_ads_ values of MTT are negative, which suggests that the adsorption is spontaneous. In addition, the values of Δ*G*^0^_ads_ range from −20~−40 KJ/mol, which indicates the adsorption belongs to a mixed physical and chemical adsorption. If the value of Δ*G*^0^_ads_ is closed to −40 KJ/mol, it is mainly chemical adsorption.

### 3.7. Theoretical Calculation

**Figure 11:**
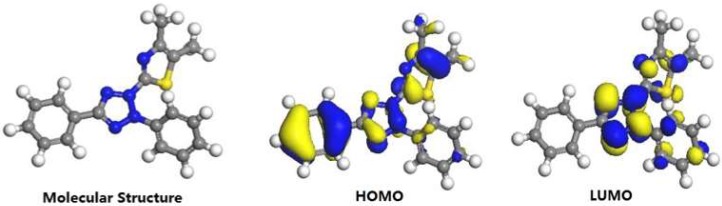
Optimized geometric structure and the frontier molecular orbital for the MTT cation.

For the sake of investing the inhibition ability and behavior, the quantum chemical calculation is analyzed. The frontier molecular orbitals of MTT cation are displayed in Figure 11, including optimized geometric structure, the lowest unoccupied molecular orbital (LUMO), and the highest occupied molecular orbital (HOMO). Meanwhile, homologous parameters are obtained and analyzed. On the basis of the frontier orbital theory, the *E*_HOMO_ is often related to the ability of donating electrons, while the *E*_LUMO_ is associated with the electron-accepting ability [52,53,54]. In other words, the low *E*_LUMO_ (−0.259 eV) value and the high *E*_HOMO_ (−0.331 eV) value for present work mean a stronger electron-donating tendency. It suggests the MTT molecules more easily donates electron to copper to form a chemical bond. The higher dipole moment (*μ* = 20.50 Debye) values and the lower energy gap values (Δ*E* = 0.072 eV) of MTT reflect the higher inhibition efficiency [55,56]. In Figure 11, the HOMO and LUMO are mainly located at whole ring, suggesting whole molecular plane as active sites are absorbed on copper surface to form the protective film.

**Figure 12:**
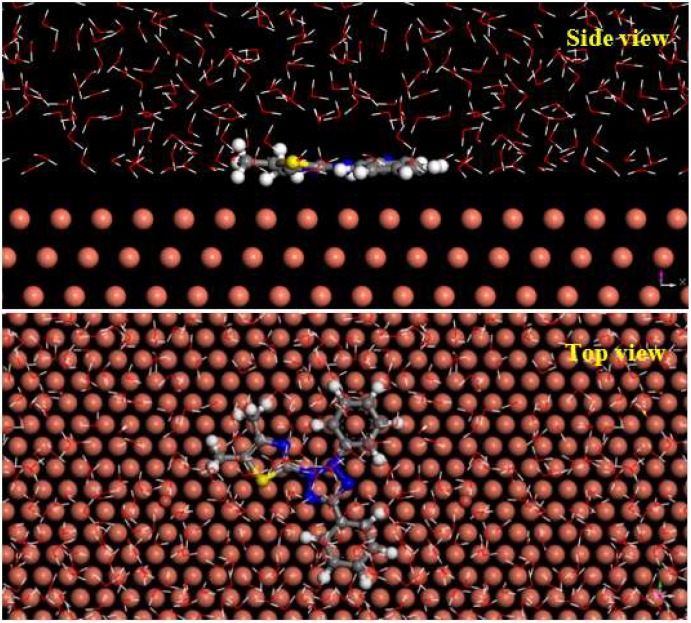
The equilibrium configuration of the molecular dynamics simulation for the MTT cation (**side view** and **top view**).

The equilibrium configurations of MD (top view, side view) of MTT cation on copper surface are shown in Figure 12. Obviously, the MTT cation is adsorbed on copper surface by a parallel mode. This approach minimizes the copper area exposed to corrosive solution, showing good inhibition performance. The active sites from quantum chemical calculation also provide relevant evidence, thus showing perfect inhibition performance. For this adsorption, it is possible that the N and S atoms from MTT provide lone pair electrons to the empty d orbitals of copper forming the coordination bond. In addition, the lower interaction energy (*E*_Cu-inhibitor_) value of −269.03 kcal mol^−1^ shows that MTT cations are strongly adsorbed on the surface of copper [57]. These results agree with experimental data.

**Figure 13:**
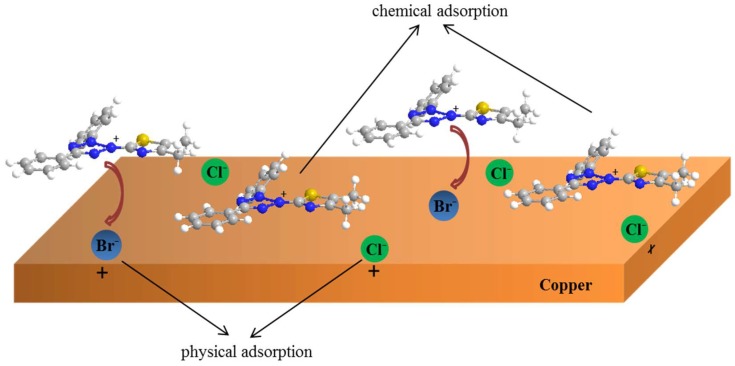
The corrosion inhibition mechanism diagram of MTT for copper in 3% NaCl solution.

On the other hand, the halide ions (Cl^−^, Br^−^, I^−^) have synergistic effects with some organic compounds for metal corrosion inhibition, according to previous reports [58,59]. Based on theoretical and experimental research, the inhibition mechanism of MTT in this study can be described, and is shown in Figure 13. Firstly, the negative bromine ions and chlorine ions are physically adsorbed onto the copper surface, and the MTT cations are adsorbed successively on copper surface by electrostatic interactions. This is thought to be physical adsorption. The adsorption of bromine and chlorine ions makes the copper surface positively charged. MTT cations with heteroatoms (N and S) form covalent bonds with copper easily, which is chemical adsorption. The FT-IR and XPS analysis concludes that the N and S atoms form covalent bonds with Cu, and the MD also provides relative evidence about adsorption, while the bromine element is not detected on the copper surface. This may be due to the fact that these ions are physically adsorbed onto the copper surface, or in small quantities.

## 4. Conclusions

Theoretical and experimental methods were applied to research the inhibition ability and inhibition mechanism. The following conclusions are obtained:

(1) The electrochemical tests, SEM, and AFM measurements demonstrate that the MTT as a mixed-type inhibitor can prevent copper corrosion effectively, and is efficiency increased with the addition of the MTT concentration.
(2) The MTT molecules form metal complex film by N and S atoms to inhibit corrosion from FT-IR spectra and XPS spectra.
(3) Adsorption isotherm studies demonstrated that adsorption for this work was a spontaneous mixed physical and chemical adsorption which obeyed Langmuir adsorption isotherm.
(4) The theoretical calculations reflected that MTT molecules processed a stronger adsorption on copper surface by a parallel mode, occupying the active site to the greatest extent by hydrophobic film, and thus, showing excellent inhibition effect.

## Figures and Tables

**Table 1:** The related parameters of EIS for copper electrode in 3% NaCl at 298 K.

	*C* (mM)	*R*_f_ (kΩ cm^2^)	*R*_ct_ (kΩ cm^2^)	*R*_p_ (Ω cm^2^)	*Q*_1_		*Q*_2_		*W* × 10^−3^ (Ω cm^2^)	*η* (%)
					*n*_1_	*C*_f_ (μF cm^−2^)	*n*_2_	*C*_dl_ (μF cm^−2^)		
Blank	0	0.095	1.970	2.065	1	8.82	0.57	681.5	1.57	–
MTT	0.05	0.021	8.675	8.696	1	8.22	0.56	601.7	–	76.25
	0.2	0.104	13.260	13.364	0.88	2.57	0.60	106.3	–	84.55
	0.5	0.133	17.310	17.443	0.94	0.89	0.57	116.8	–	88.16
	1	0.089	21.580	21.669	0.94	0.96	0.59	213.8	–	90.47
	5	0.382	47.990	48.372	0.99	0.38	0.52	123.8	–	95.73

**Table 2:** The relevant parameters from polarization curve measurement.

	*C* (mM)	*E*_corr_ (mV)	*I*_corr_ (A cm^−2^)	*β*_c_ (mV dec^−1^)	*β*_a_ (mV dec^−1^)	*η* (%)
Blank	0	−186	4.124 × 10^−6^	−167.2	59.4	–
MTT	0.05	−144	1.073 × 10^−6^	−149.7	78.4	73.98
	0.2	−168	1.010 × 10^−6^	−159.2	78.2	75.51
	0.5	−176	5.877 × 10^−7^	−138.1	128.7	85.75
	1	−181	4.559 × 10^−7^	−149.1	124.5	88.95
	5	−186	3.184 × 10^−7^	−117.7	118.4	92.28

**Table 3:** The homologous binding energy, chemical states and FWHM XPS spectra peaks from the surface of the blank and Cu-MTT film, respectively.

		The Blank			Cu-MTT	
	Chemical State	Binding Energy (ev)	FWHM	Chemical State	Binding Energy (ev)	FWHM
C1s	C–C/C–H	284.31	1.15	C–C/C–H	284.39	1.20
	C–O–C	286.16	1.15	C=N/C–S	285.62	1.20
	O–C=O	287.70	1.15	C–N	286.61	1.20
				O–C=O	287.71	1.20
Cu2p	Cu(0)/Cu(I)	931.90	1.13	Cu(0)/Cu(I)	931.95	1.7
				CuO	933.80	1.7
O1s	CuO/Cu_2_O	530.14	1.00	CuO/Cu_2_O	530.65	1.80
	O–C=O	531.30	1.00	O–C=O	531.98	1.80
	C–O–C	532.10	1.00	N=N	398.45	1.00
N1s				N=N	398.45	1.00
				N-N	399.20	1.00
				C=N	400.10	1.00
				C–N	400.70	1.00
				N:Cu	401.77	1.00
S2p				S–C	163.75	1.40
				S:Cu	165.07	1.40

**Table 4:** The relevant thermodynamic parameters for copper from Langmuir adsorption isotherm.

Measurements	*K*_ads_ (×10^3^ L/mol)	Δ*G*^0^_ads_ (KJ/mol)
Polarization	29.41	−35.44
EIS	29.07	−35.41

## Supplementary Materials

The following are available online at http://www.mdpi.com/1996-1944/11/6/1042/s1, Figure S: The impedance diagram for copper electrode in 5 mM MTT solution with different immersion time at 298 K.
