# Supplementary material for: Experimental and Theoretical Investigation of Thiazolyl Blue as a Corrosion Inhibitor for Copper in Neutral Sodium Chloride Solution
Source: Materials (Basel). 2018 Jun 19;11(6):1042. doi: 10.3390/ma11061042 (PMC6025645; doi:10.3390/ma11061042)
Supplement: Supplementary file 1 [file materials-11-01042-s001.pdf]

materials

Supplementary Material

Experimental and Theoretical Investigation of Thiazolyl Blue as a Corrosion Inhibitor for Copper in Neutral Sodium Chloride Solution

Li Feng [1], Yujie Qiang [1], Shengtao Zhang [1,2,*], Yue Xu [1], Lei Guo [3,*] Loutfy H. Madkour [4] and Shijin Chen [5]

1 School of Chemistry and Chemical Engineering, Chongqing University, Chongqing 400044, China; fengli_cqu@163.com (L.F.); yqiang_cqu@163.com (Y.Q.); cqtanjc@163.com (Y.X.)
2 Material Corrosion and Protection Key Laboratory of Sichuan province, Zigong 643000, China
3 School of Material and Chemical Engineering, Tongren University, Tongren 554300, China
4 Chemistry Department, Faculty of Science and Arts, Al Baha University, Baljarashi 65635, Saudi Arabia; loutfy_madkour@yahoo.com
5 Bomin Electronics Ltd., Meizhou 514021, China; fwjshen@163.com (S. C.)
* Correspondence: stzhang@cqu.edu.cn (S.Z.); cqglei@163.com (L.G.)

Received: 24 May 2018; Accepted: 13 June 2018; Published: 19 June 2018

Durability test

For all electrochemical tests, immersing and testing are conducted in 3% NaCl solution with and without MTT. The OCP potential reaches a steady state for1800s, suggesting the protective film can be formed in a short time. Furthermore, the durability measurement was performed to explore the durability of the Cu-MTT film. Figure S shows the impedance diagram for copper electrode in 5 mM MTT solution with different immersion time at 298 K. The inhibition effect increases when the immersion time is less than 3 h (95.7% for 0.5 h, 96.4% for 1 h, 96.8% for 2 h, 97.1% for 3 h), which is due to the rearrangement of adsorbed layer. The efficiency decreases slightly when the immersion time is more than 3 hours (97.1% for 4 h, 97.05% for 5 h, 91.8% for 10 h), which is owing to the desorption of film and unsound film. It can be seen that the film is stable in a short period of time (<5 h). However, when the immersion time is more than 10 h, the protective effect of the film becomes worse. This is due to corrosive substances in the solution such as Cl^- or unsound film.

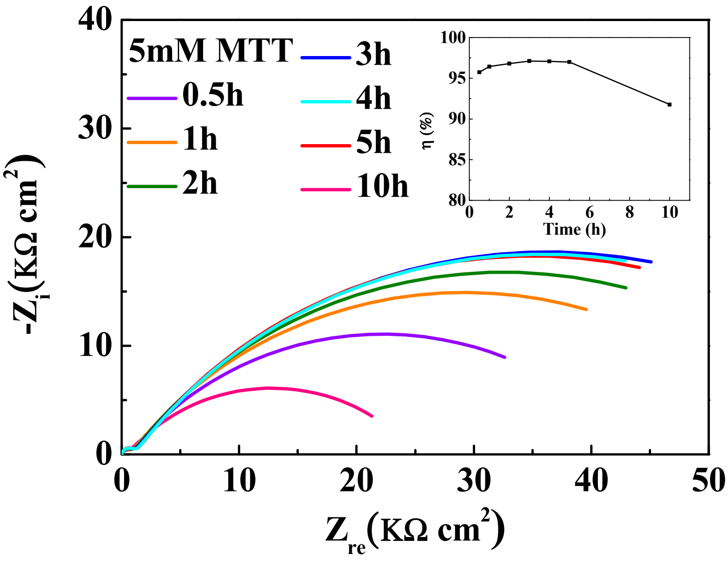

Figure S. The impedance diagram for copper electrode in 5 mM MTT solution with different immersion time at 298 K.
